# The patient-driven Rare Disease Research Network: turning research on its head

**DOI:** 10.1136/bmjopen-2025-105045

**Published:** 2025-10-27

**Authors:** Jo Balfour, Laura B Cowley, Georgina Windsor, Ellie Dalby, Miles Sibley, Amy Hunter, Rona M Smith

**Affiliations:** 1 Cambridge Rare Disease Network, Cambridge, UK; 2 Department of Medicine, University of Cambridge, Cambridge, UK; 3 Patient Led Research Hub, Cambridge, UK; 4 Rare Disease Research Network, Cambridge, UK; 5 Babraham Research Campus, Cambridge, England, UK; 6 Patient Experience Library, Ballymoney, Northern Ireland, UK; 7 Genetic Alliance UK, London, London, UK; 8 Cambridge University Hospitals NHS Foundation Trust, Cambridge, UK

**Keywords:** Patient Participation, Community-Based Participatory Research, Digital Technology, Health Equity, Organisational development, Rare Diseases

## Abstract

**Background:**

The vast majority of healthcare research in the UK is investigator-led. While national progress in patient and public involvement (PPI) increasingly mandates patient consultation, research questions and outcomes still frequently misalign with patient priorities. This is particularly important in rare disease research, as more than 95% of 11 000 conditions have no effective or curative treatment, and around 20% are not clinically defined, making them difficult to diagnose and manage. The unmet physical, mental and emotional needs of people living with rare diseases are immense. Extensive guidance and toolkits exist to support investigators with PPI, but none target patient communities attempting to promote their own priorities, initiate or co-lead research.

**Aim:**

This communication article introduces the newly established patient-led Rare Disease Research Network (RDRN).

**What is the RDRN, and how can it be useful?:**

Launched in November 2024, the RDRN is an open-access collaborative platform designed to support patient-driven and co-produced research, connecting patient and professional partners with similar research interests. Originally conceived by an ultra-rare patient group, the network was co-produced with the rare disease community, including individuals living with rare conditions, parents, carers and charity advocates, whose lived experience and priorities shaped every aspect of its design. Supported by academic and research networks, its collaborative development ensures RDRN removes barriers to participation while complementing existing initiatives. RDRN is a novel approach to driving new impactful research by aligning investigator priorities with real-world needs and building capacity from patients outward. Rare disease communities bring lived expertise, creativity and motivation. Yet without a structured route to collaborate, their insights are often lost. RDRN offers an inclusive space, fostering new partnerships and supporting upstream collaboration. The approach enables patients to become ‘research ready’ and empowers them to have an active role in generating ideas and delivering research from inception, leading to innovative research and driving meaningful change in patients’ lives. With further development, RDRN could present a lasting, scalable and unified model for co-designed rare disease research. By enabling trust, capacity and shared purpose, it can drive discovery, improve outcomes and build a more resilient and self-sustaining research ecosystem, underpinning key pillars of the 2021 UK Rare Diseases Framework.

**KEY MESSAGES**

- Rare disease research is difficult because there are few patients, few clinical experts, fragmented care and limited funding. For rare disease research to be effective and efficient, it must be designed with patient partners to ensure it is acceptable and accessible to participants.
- Most studies are patient-centred but almost invariably investigator-led. There is no clear pathway or support for patient communities to propose and develop their own research ideas.
- The Rare Disease Research Network is a new online platform aimed at supporting patient-driven research, providing resources, mentorship and matchmaking with investigators.

## UK rare disease research landscape

There are nearly 11 000 rare diseases,^1^ each affecting fewer than one in 2000 people with a combined impact of over 3.5 million people (1 in 17) affected in the UK.^2^ Over 95% of rare diseases lack effective and curative treatments^3^ and up to 20% are not clinically defined,^2^ significantly impacting diagnosis, treatment and patient well-being. The unmet needs of people living with rare diseases are immense, compounded by multi-organ involvement, limited specialist expertise and fragmented care. Research is critical to improving diagnostic accuracy, advancing treatment development and understanding patient experiences.

The 2021 UK Rare Diseases Framework,^2^ associated action plans from devolved nations^4^,^7^ and 2024 initiatives including the Rare Disease Research UK (RDR UK)^8^ and LifeArc’s Translational Centres for Rare Diseases^9^ have created significant momentum for cross-sector collaboration and patient involvement in rare disease research. Between 2016 and 2021, over one billion pounds was invested by the Medical Research Council (MRC), National Institution for Health and Care Research (NIHR), Association of Medical Research Charities (AMRC) and industry partners into rare disease project grants and fellowships across the UK.^10^ However, despite this substantial amount of money, more than 9000 rare conditions—over 80%—received no research investment, with funding heavily concentrated around relatively few, better-known rare conditions.^10^ An internal 2024 follow-up exercise by LifeArc confirmed the trend: national investment is still clustered around single-disease translational initiatives, and these projects are not easily accessible to grassroots patient groups. Major gaps remain in the rare disease research ecosystem—particularly around upstream partnership development and support for patient-driven research ideas.

In parallel to rare disease monetary investment, there have been major advances in trial innovation in rare diseases, including the use of adaptive designs*, Bayesian methodologies* and master protocols*, which have introduced both new opportunities and challenges. One feature of these methods is collecting all relevant existing data, however limited, and making prior assumptions. These methods hold promise for accelerating research in ultra-rare conditions and heterogeneous populations, but their success depends on meaningful patient involvement. Co-developing study designs with patient partners can surface critical insights around acceptability, trial burden and relevance, not just of the research question but of any prior assumptions made. Yet for this to happen, patients must first be supported to fully understand these complex concepts and methods to be able to meaningfully contribute to research design choices—a process that requires intentional, tailored resources and facilitation.

## Patient-centred but not patient-driven

While healthcare research is increasingly patient-centred, it remains largely investigator-led, with practical and cultural barriers to genuinely co-produced or patient-driven research. Major funders now mandate a compelling patient and public involvement (PPI) plan, and some support patients and charities as co-applicants, but these efforts rarely enable equitable partnerships. A range of challenges, including organisational culture, bureaucracy, additional administration workload, investigator reluctance or uncertainties, lack of upstream funding,^11^,^13^ as well as the absence of required PPI reporting throughout funded research, means that efforts can be transient and tokenistic. Even motivated investigator-led research teams often lack the capacity and tools to involve lived experience from ideas’ inception, as these efforts are deemed time-consuming and ‘risky’. Furthermore, the current structure for academics in the UK poses challenges, as PPI is a low priority for career progression and short-term contracts do not allow time to embed trusted relationships with patient communities. These barriers are particularly stark in rare diseases, where dispersed patient populations and small support groups often face additional access and resource challenges.

Nonetheless, patient communities remain highly motivated to engage, contribute to investigator-led proposals and help shape the UK research agenda. While there are a multitude of PPI initiatives, there is a persistent gap in services available for individuals and small charities keen to progress their own ideas into viable studies. The Patient Led Research Hub (PLRH)^14^ is the only UK initiative designed to support rare disease patient groups to develop and deliver their own proposals. Its novel approach provides patient groups with a clear pathway to accessing publicly funded research support services designed to support academic investigators. The programme has shown success in surfacing impactful, peer-reviewed and competitively funded research that addresses real-world unmet needs. However, as a collaboration between Cambridge NIHR Biomedical Research Centre and the University of Cambridge, the PLRH is bound by remit and organisational constraints. It does not have the capacity to offer bespoke training for patient groups to become research ready or for investigators to develop skill sets imperative to co-production.

## Wasted resources

In a crowded landscape of investigator-led research and PPI initiatives, waste and poor value for money can be highlighted in four key areas:

### Poor primary objectives

Unsurprisingly, a 2022 review of randomised clinical trials, which included rare diseases, confirmed that trial teams typically decide the primary research outcome.^15^ However, when published trials were shown to patients and healthcare professionals with experience of the conditions being studied, just 28% agreed with the choice of primary outcome. The authors state, “The kindest thing that can be said about this is that it represents research waste. Less kindly, it means patients and healthcare staff have spent their time, energy, goodwill and perhaps hope on a trial that has failed to provide the key information that people like them need in order to make better treatment decisions.”

Patients frequently identify unmet needs, overlooked questions or real-world observations that could generate powerful hypotheses. For example, patients with multiple conditions may have insights into how a medication prescribed for one condition appears to help or worsen another, sparking a testable mechanistic research question. These types of questions, while often invisible to traditional research prioritisation systems, can lead to meaningful, cross-disease learning when captured and supported early.

### The toolkit mountain

There is a paucity of support for the earliest stages of co-production before partnerships are established and only serendipitous pathways for patient groups to collaborate with open-minded investigators. Existing PPI and research support services either presume patient partnerships have been established or focus on barriers that investigators face when delivering PPI.

For example, in the UK, NIHR James Lind Alliance Priority Setting Partnerships^16^ include patients and advocates within their multi-stakeholder approach to identifying research priorities, but they do not facilitate research conduct and there is no mechanism for patient communities to remain involved once a priority is taken forward by investigators. Alternatively, University College London’s Co-Production Collective^17^ champions co-production, but focuses on qualitative and secondary research.

There is a plethora of guidance for investigators on how to access and involve patients and the public in their research and some insight on the principles of co-production. Over 550 PPI toolkits (the ‘toolkit mountain’) aiming to support investigators have been catalogued across the UK: between 2016 and 2020 these were produced at the rate of one a week.^18^ Although such guidance can be useful and co-written with patient partners, it typically remains focused on facilitating patient contributions to investigator-led research. Moreover, not one of the 550 toolkits reviewed had a specific focus on involvement or engagement with rare disease communities. A substantial gap remains for patient groups seeking to initiate research, become ‘research ready’ to confidently co-produce research with investigators or support communities who experience epistemic injustice* and challenges to their perceived representativeness.

### Duplication of effort

Internal evidence mapping conducted by the Patient Experience Library in 2022 revealed extensive duplication in research investigating people’s experiences of care. For example, 35–50% of reviews on experiences of digital healthcare, COVID-19, urgent and emergency care and healthcare for people experiencing homelessness focused on access to services. Over half of the studies involving rare disease communities focused on access and communication. While it is vital to understand good practice and barriers to access, trust is quickly eroded when services ask users repeatedly for feedback on issues that remain largely unchanged. Such duplication does not add novel insight, and effort should instead be placed on collaborative working and knowledge sharing to ensure widespread, transferrable benefit across healthcare services.

### The PPI paradox

National progress in PPI has led to many funders mandating the inclusion of a designated PPI Lead on all applications while, paradoxically, not equipping or supporting them to fulfil the role requirements. A 2024 review notes that ‘the specifications, skills and support needs (of a PPI Lead) have yet to crystallise into a distinct career trajectory.^19^’ The result is that PPI roles are ‘typically underfunded with poor job security’, they ‘do not confer prestige’ and ‘many PPI workers describe their work as ‘invisible’’.^19^ In addition, some funders do not support patient charities as co-applicants, denying fair opportunity and recognition of their value in healthcare research from the outset.

## Establishing the Rare Disease Research Network

A unique opportunity arising from these challenges is to build capacity from the rare disease community outward and support lived experience as a driver of healthcare research. Since 2023, PLRH and Cambridge Rare Disease Network (CamRARE) have been working to resolve the disconnect between patients and investigators, equipping patient groups with the skills, networks and confidence to engage as equal partners from the outset of the research lifecycle.

Originally funded by an 18-month NIHR ‘Public Partnership’ grant, the Rare Disease Research Network (RDRN) was co-produced through a series of structured activities between 2023 and 2024. Over 20 contributors from rare disease communities, advocacy organisations and patient support groups were selected through an open call and targeted invitations to ensure diverse representation across conditions, geographies and research experience. An inclusive, agile management approach enabled members to participate in all decision-making, including finance and vendor appointments. In-person workshops and monthly online consultations directly informed all aspects of the platform’s user interface and functionality; individuals were encouraged to contribute in a manner that best suited their needs, with appropriate accommodations and regular touchpoints for those requiring more time, different formats or time away due to ill health or carer’s commitments. Additional outreach with academic investigators, research organisations, funders and industry groups from across the UK ensured RDRN’s vision and function complemented investigator-led and PPI initiatives without duplication.

The resultant novel, open-access platform (https://rd-rn.org) reflects the priorities, language and lived experiences of its user community. It enables patient communities to share and shape research ideas. Tailored resources, peer support and mentorship opportunities help communities become ‘research ready’ and equipped with the skills and resources required to co-develop research ideas, whether initiating their own projects or collaborating on investigator-led proposals. New patient-driven research ideas can be showcased and supported throughout the research journey, from inception, establishing a multi-stakeholder team, through to submitting competitive external grant applications. It allows investigators and research services to readily access motivated patient communities, supporting the involvement of end-users from the outset, ensuring that innovations and outcomes align with real-world needs while driving more ethical and accessible research. New partnerships are facilitated and moderated by an RDRN team with a diverse range of professional backgrounds and lived experiences.

RDRN launched in November 2024 and within 6 months established a membership of nearly 250 individuals from stakeholders across the globe. The platform’s value has been demonstrated in pilot activities including online idea submissions, peer support and matchmaking with academic and industry partners. These activities confirm the willingness of patients to drive research, the demand for a structured entry point into the system and the importance of creating a trusted space for upstream collaboration vital for research development. Further work is now required to establish a sustainable business model which effectively captures impact, reach and the benefits of patient-driven research.

## Future direction and barriers

Feedback from early RDRN adopters demonstrates a strong desire for its model to evolve into a permanent, sustainable infrastructure that facilitates high-quality research aligning patient priorities with national strategies and action plans. Wide-ranging support in principle from public and charitable organisations across the UK, including NIHR, RDR UK, LifeArc and healthcare and research support services, has the potential to build capacity, enable consistent cross-sector communication, shared learning and streamlined stakeholder engagement. However, despite the clear unmet need and appetite for new infrastructure, practical support and investment remain lacking. RDRN’s approach to co-production, permitting time and space to foster trust and accessibility, has been deemed by some funders to be inefficient and unnecessary within the current PPI landscape.

With limited resources, RDRN’s current focus remains centred on upskilling patient communities via tailored resources and mentorship. Research networks associated with PLRH and CamRARE continue to provide pro bono support to help patients refine and develop their research ideas, but further commitment is also needed from open-minded professionals and organisations to create an in-house consultation team, ready to share their expertise in research design, delivery and clinical care. However, competing demands, limited capacity and lack of PPI recognition within career progression are real barriers to sustained professional involvement and must be mitigated. Ideally, as further investment is secured, RDRN can expand its services to support and train research professionals, helping to de-risk patient-driven and co-production methodologies, promoting them as best practice and encouraging investigator uptake.

## Conclusion

RDRN has the potential to drive meaningful change in patient care by facilitating upstream partnerships with motivated, knowledgeable patient communities. Its novel approach to innovative research, both cross-cutting and rare disease-specific, can help underpin the UK Rare Disease Framework, while learnings can benefit wider efforts where upstream patient partnerships are vital.

## Glossary

*Adaptive designs*: clinical trial designs which allows changes to be made to one or more aspects of the trial while the research is ongoing.

*Bayesian methodologies*: statistical approach useful for rare diseases where little is known, as existing data (e.g. routinely collected healthcare data) can be used to make predictions about trial outputs.

*Epistemic injustice*: devaluing or excluding knowledge because of social biases or prejudices; for example, patients’ experiences not being taken seriously because ‘doctors’ know best.’

*Master protocol*: clinical trial which uses one overarching protocol to evaluate multiple interventions or diseases at the same time.
