# Supplementary material for: The patient-driven Rare Disease Research Network: turning research on its head
Source: BMJ Open. 2025 Oct 27;15(10):e105045. doi: 10.1136/bmjopen-2025-105045 (PMC12570899; doi:10.1136/bmjopen-2025-105045)
Supplement: Supplementary file 1 [file bmjopen-2025-105045.reviewer_comments.pdf]

PEER REVIEW HISTORY

BMJ Open publishes all reviews undertaken for accepted manuscripts. Reviewers are asked to complete a checklist review form and are provided with free text boxes to elaborate on their assessment. These free text comments are reproduced below.

ARTICLE DETAILS

Title (Provisional)

The patient-driven Rare Disease Research Network: turning research on its head

Authors

Balfour, Jo; Cowley, Laura B; Windsor, Georgina; Dalby, Ellie; Sibley, Miles; Hunter, Amy; Smith, Rona M

VERSION 1 - REVIEW

Reviewer 1
Name Bathen, Trine
Affiliation Sunnaas Hospital TRS Competence Center for Rare Diseases
Date 08-Aug-2025
COI I have co-authored one of the articles I have suggested as a possible reference - Velvin et al 2022. https://ojrd.biomedcentral.com/articles/10.1186/s13023-022-02357-y

---

This article addresses an important topic and gives a good overview on current practice in the UK. I have some minor suggestions on changes that might make the article easier to follow also for readers without insight to the UK rare disease research community. This concerns mainly the use of very many acronyms that non- UK readers will not know. I also think it might be useful to use some of recent international research on patient involvement in research to underline some of your arguments. Some suggestions are given in attached document, in comments to each section.

The references used in the article are updated and appropriate for Patient partner involvement in research as far as I can assess. I would however, suggest also cheking if there are relevant non UK studies on patient involvment in rare disease research that also could be used to strenghten your arguments and enhance the relevance of the article for the non UK readers.

Response to authors:

This article addresses an important topic and gives a good overview on current practice in the UK. I have some minor suggestions on changes that might make the article easier to follow also for readers without insight to the UK rare disease research community. This concernes mainly the use of very many acronyms that non- UK readers will not know. I also think it might be useful to use some of recent international research on patient involvement in research to underline some of your arguments. Some suggestions are given below in comments to each section.

**Section**	**Response/ suggestions**
Abstract	Although this is not a research article I think some headlines would make it easier to follow for the reader a short aim in the beginning of the abstract. For instance, start with Aim: the aim of this article is to present the Rare disease Research network...., then background, and lastly: What is RDRN and how can it be useful
Main text<br>UK rare disease research landscape	The main text has a logical structure. Explaining the UK rare disease research landscape first, which is interesting and recognisable also for a non- UK reader.<br>- I have a question about the statement in the end of line 7, page 2 (bottom right corner)- is this your experience or do you have a reference for this statement? Please clarify.<br>- The same goes for the statement on line 10 about the 2024 follow-up by LifeArc were a reference perhaps also excist?<br>- In the paragraph starting on line 18 page 2, you mention terms that you then explain in the glossary in the end. For the reader I think it would be more understandable if you perhaps incorporated this in the text.
Main text<br>Patient centered but not patient driven	I think you are right in the assumption that you make in line 35, page 2 that healthcare research is incresingly patient-centered but still is mainly investigator led. I think you would strengthen the article and underpin your arguments if you also used research references here - Like for instance:<br>-How are patient partners involved in health service research? A scoping review of reviews | Research Involvement and Engagement | Full Text<br>-Patient involvement in rare diseases research: a scoping review of the literature and mixed method evaluation of Norwegian researchers' experiences and perceptions | Orphanet Journal of Rare Diseases | Full Text
Main text<br>Wasted resources	The section describes very interesting and important topics, my main comment is to suggest that you consider also using research on patient involvement in rare diseases as references, as I think most of the references you have used in this section is about patient involvement in general.<br><br>I have some comments to The toolkit mountain. Line 40 page 3:<br>- The James Lind Alliance – I suggest including this also as a reference with link to the webpage. You also state in line 45,

	that there is no mechanism for patient involvement to remain involved with research development once commissioned or allocated to an investigator team – for a reader who does not know the James Lind Alliance that well this was a bit difficult to understand – can you please explain why?<br>- University College Londons Co-Production Collective – should perhaps also be added to the reference list, like other initiatives you have listed.<br>- Would it here also be relevant to mention the resource from the European Joint Programe on Rare diseases? SHORT-GUIDE-ON-PATIENT-PARTNERSHIPS-IN-RARE-DISEASE-RESEARCH-PROJECTS.pdf
Main text<br>Establishing the rare diseases research network	This section is clear and easy to understand. However, you might explain in more detail how you plan to evaluate the effect of the Rares diseases research network portal, as this might be a good way to meet the current research gap that exists in defining and measuring patient partner involvement highlighted by both Tcherning et al 2025 and Velvin et al 2022.
Main text<br>Future direction and barriers	-This section I think is hard to read because of the extensive use of acronyms, consider writing the full names of the listed institutions.<br>-Line 41 page 3, statement that some funders deem the RDRN'S approach inefficient and unnecessary – here I at least get curious, is this your experience, is it an extensive problem? How many and why? – if you have space inside article word count perhaps specify a little more?<br><br>- On page 2, line 47 you explain that one major obstacle for patient partner involvement is the structure for academics in the UK (and probably not only the UK) – is this a point you could discuss more in future directions? – do you have any thoughts on or if the RDRN can influence this?<br>- On page 6 – the last paragraph – could this be named Conclusion?

**Reviewer**	**2**
**Name**	**Talarico, Rosaria**
**Affiliation**	**University of Pisa, Rheumatology Unit**
**Date**	**08-Sep-2025**
**COI**	**None**

The present article entitled 'The patient-driven Rare Disease Research Network: Turning research on its head' is aimed at considering the UK rare diseases research landscape and the wasted resources.

I believe that the content is very important. I would suggest:

*Adding some consideration regarding other rare diseases network that have been working with this challenges in rare diseases (e.g. European Reference Networks);

*Identifying 2-3 main key messages that can guide the readers in the lecture of the text.

VERSION 1- AUTHOR RESPONSE

Reviewer 1

1. Abstract: Although this is not a research article I think some headlines would make it easier to follow for the reader a short aim in the beginning of the abstract. For instance, start with Aim: the aim of this article is to present the Rare disease Research network...., then background, and lastly: What is RDRN and how can it be useful.
   We have incorporated abstract subheadings as advised.

2. Main text: UK rare disease research landscape
   a. The main text has a logical structure. Explaining the UK rare disease research landscape first, which is interesting and recognisable also for a non- UK reader.. I have a question about the statement in the end of line 7, page 2 (bottom right corner)- is this your experience or do you have a reference for this statement? Please clarify.
      This statement is taken from the Rare Diseases Research Landscape Project Report (p19: “Most rare diseases listed in Orphanet had zero awards associated with them in the NIHR Programmes and MRC rare disease research portfolio (n = 9,278).”) We have added the appropriate reference.

   b. The same goes for the statement on line 10 about the 2024 follow-up by LifeArc where a reference perhaps also exists.
      As an internal LifeArc exercise, there is no reference to link with this statement.

   c. In the paragraph starting on line 18 page 2, you mention terms that you then explain in the glossary in the end. For the reader I think it would be more understandable if you perhaps incorporated this in the text.

After consulting with lay-readers, we have chosen to keep the glossary at the end of the manuscript. This is because definitions are lengthy and would interrupt the flow of the main text. Further, these definitions are tangential to the purpose of manuscript, and understanding the detail is not necessary to understand the manuscript itself.

3. Main text: Patient centred but not patient driven: think you are right in the assumption that you make in line 35, page 2 that healthcare research is increasingly patient-centred but still is mainly investigator led. I think you would strengthen the article and underpin your arguments if you also used research references here - Like for instance: How are patient partners involved in health service research? A scoping review of reviews & Patient involvement in rare diseases research: a scoping review of the literature and mixed method evaluation of Norwegian researchers' experiences and perceptions
   Thank you for highlighting these articles; we have included them to illustrate challenges and misconceptions of PPI in healthcare research.

4. Main text: Wasted resources
   a. The section describes very interesting and important topics, my main comment is to suggest that you consider also using research on patient involvement in rare diseases as references, as I think most of the references you have used in this section is about patient involvement in general.
      Thank you for raising this point. We have clarified that the 2022 review ('poor primary objectives' section) included rare diseases. In the 'toolkit mountain' section, we clarify that not one toolkit focuses on rare disease communities. In 'duplication of effort' we have added a sentence specific to rare disease reviews.

   b. The toolkit mountain. Line 40 page 3: The James Lind Alliance – I suggest including this also as a reference with link to the webpage. You also state in line 45, that there is no mechanism for patient involvement to remain involved with research development once commissioned or allocated to an investigator team – for a reader who does not know the James Lind Alliance that well this was a bit difficult to understand – can you please explain why?
      We have added a reference for the James Lind Alliance (JLA). The JLA is commissioned to facilitate multi-stakeholder activities to develop a 'top 10' list of research priorities. Once complete, the list is shared publicly and sometimes used by funders to direct funding calls. Individual contributors are not named, and the JLA does not track research associated with these lists. As such, there is no way for a patient advocate to suggest an unmet need or propose a research idea within JLA activities and also be involved in

the development or conduct of the research. We have tried to clarify this in the text.

c. University College London's Co-Production Collective – should perhaps also be added to the reference list, like other initiatives you have listed.
We have added a reference for the Co-Production Collective.

d. Would it here also be relevant to mention the resource from the European Joint Programme on rare diseases? SHORT-GUIDE-ON-PATIENT-PARTNERSHIPS-IN-RARE- DISEASE-RESEARCH-PROJECTS.pdf
Thank you for highlighting this resource. Although co-written with patient partners and including useful case studies, it still focuses on investigator-led research and is primarily designed for investigators. It does not guide patient groups on how to propose, approach investigators, or develop their own ideas. We have adapted the 'toolkit mountain' section to clarify the unmet need is specific to patient-driven research, not consultation-based PPI.

5. Main text: Establishing the rare diseases research network: This section is clear and easy to understand. However, you might explain in more detail how you plan to evaluate the effect of the Rare Disease Research Network portal, as this might be a good way to meet the current research gap that exists in defining and measuring patient partner involvement highlighted by both Tcherning et al 2025 and Velvin et al 2022.
Evaluating the reach and impact of RDRN is important, however we have not yet agreed the process to do so. NIHR-funded activities focused on building partnerships with the rare disease community and co-creating the RDRN platform. We are currently seeking additional funding to, in part, develop a reliable and representative means of measuring RDRN impact. In the ethos of co-production, this will be an inclusive and iterative approach which is not yet defined, but we now reference this aspiration within the main text.

6. Main text: Future Direction and barriers:

e. This section I think is hard to read because of the extensive use of acronyms, consider writing the full names of the listed institutions.
These acronyms are expanded when first introduced (paragraph 2 of main text).

f. Line 41 page 3, statement that some funders deem the RDRN's approach inefficient and unnecessary – here I at least get curious, is this your experience, is it an extensive problem? How many and why? – if you have space inside article word count perhaps specify a little more?

We have struggled to receive follow-on funding to develop RDRN from both regional and national funders. We have consciously decided to not go into detail in this section to not alienate future efforts, but also because we are aware that some of these funders are currently undergoing an organisational shift in remit in response to our feedback.

g. On page 2, line 47 you explain that one major obstacle for patient partner involvement is the structure for academics in the UK (and probably not only the UK) – is this a point you could discuss more in future directions? – do you have any thoughts on or if the RDRN can influence this?
   With further funding we hope to expand RDRN to help mitigate some of the challenges experienced by academic researchers. We have added some text to clarify this aim, however, as with measuring RDRN impact, the exact process will again be iterative and co-produced and thus we cannot be prescriptive about deliverables.

h. On page 6 – the last paragraph – could this be named Conclusion?
   'Conclusion' header has been added.

Reviewer 2

I believe that the content is very important. I would suggest:

7. Adding some consideration regarding other rare diseases networks that have been working with this challenge in rare diseases (e.g. European Reference Networks).
   We recognise, and collaborate with, many organisations across the globe aiming to improve patient involvement and engagement with rare disease communities. Some initatives (eg Global Genes, Beacon for Rare Diseases) also providing research training and mentorship for patient groups. However, to our knowledge, the RDRN is the first online platform aiming to facilitate patient-intiated research, enabling patient groups to develop ideas that focus on their own unmet needs, connect with relevant stakeholders, and be equal partners in progressing proposals and conducting research. We have tried to clarify this focus within the main text by differentiating between 'patient consultation' or 'traditional PPIE' and patient-driven research or co-production.

8. Identifying 2-3 main key messages that can guide the readers in the lecture of the text.
   We have added a 'key messages' section between the abstract and main text with the following points:
   1) Rare disease research is difficult because there are few patients, few clinical experts, fragmented care, and limited funding. For rare disease research to be

effective and efficient, it must be designed with patient partners to ensure it is acceptable and accessible to participants.

2) Most studies are patient-centred but almost invariably investigator-led. There is no clear pathway or support for patient communities to propose and develop their own research ideas.
3) The Rare Disease Research Network is a new online platform aimed at supporting patient-driven research, providing resources, mentorship, and match-making with investigators.

VERSION 2 - REVIEW

Reviewer 1

Name Bathen, Trine

Affiliation Sunnaas Hospital TRS Competence Center for Rare Diseases

Date 02-Oct-2025

COI

Thank you for updating the manuscript. I have no further comments.
